# Can high-flow nasal cannula reduce the rate of reintubation in adult patients after extubation? A meta-analysis

**DOI:** 10.1186/s12890-017-0491-6

**Published:** 2017-11-17

**Authors:** Yue-Nan Ni, Jian Luo, He Yu, Dan Liu, Bin-Miao Liang, Rong Yao, Zong-An Liang

**Affiliations:** 1 Department of Respiratory and Critical Care, Sichuan University, No.37 Guoxue Alley, Chengdu, 610041 China; 2 Department of Critical Care Medicine, Sichuan University, No. 37 Guoxue Alley, Chengdu, 610041 China; 3 Department of Emergency, Sichuan University, No. 37 Guoxue Alley, Chengdu, 610041 China

**Keywords:** High flow nasal cannula, Adult, Post-extubation, Meta-analysis, Mortality, Prognosis

## Abstract

**Background:**

The effects of high flow nasal cannula (HFNC) on adult patients after extubation remain controversial. We aimed to further determine the effectiveness of HFNC in comparison to noninvasive positive pressure ventilation (NIPPV) and conventional oxygen therapy (COT).

**Methods:**

The Pubmed, Embase, Medline, Cochrane Central Register of Controlled Trails (CENTRAL) as well as the Information Sciences Institute (ISI) Web of Science were searched for all the controlled study comparing HFNC with NIPPV and COT in adult patients after extubation. The primary outcome was rate of reintubation and the secondary outcomes were intensive care unit (ICU) mortality and length of ICU stay (ICU LOS).

**Results:**

Eight trials with a total of 2936 patients were pooled in our final studies. No significant heterogeneity was found in outcome measures. Compared with COT, HFNC was associated with lower rate of reintubation (Z = 2.97, *P* = 0.003), and the same result was found in the comparison between HFNC and NIPPV (Z = 0.87, *P* = 0.38). As for the ICU mortality and ICU LOS, we did not find any advantage of HFNC over COT or NIPPV.

**Conclusions:**

In patients after extubation, HFNC is a reliable alternative of NIPPV to reduce rate of reintubation compared with COT.

## Background

Mechanical ventilation is a life-saving method [1], which has been proved to improve gas exchange as well as decrease work of breathing due to fully or partially spontaneous breathing replacement. Unfortunately, invasive mechanical ventilation has been increasingly recognized to be associated with various adverse events, such as ventilator-associated pneumonia and barotrauma. Moreover, the hospital mortality of patients admitted into intensive care unit(ICU) remains as high as 30.7% [2, 3]. Timely extubation is one way of minimizing the morbidity [4]. However, it is estimated that 12 to 14% of patients who undergo planned extubation will require reintubation within 48 to 72 h, most within the first 24 h [2, 5–7].

NIPPV may prevent post-extubation respiratory failure and avoid reintubation if it is applied soon after extubation [8–12]. In addition, according to the most recent guidelines, preventive NIPPV was recommend in patients with high risk of reintubation [13]. However, numerous potential hazards, such as skin damage, eye irritation, interface intolerance, diet and expectoration interruption, might block the usage of NIPPV in clinical practice [14]. Thus, potential substitutions of NIPPV without the adverse events mentioned above are imperatively needed.

High-flow nasal cannula (HFNC) is a new technique of oxygen delivering, which delivers heated and humidified oxygen via special devices at a rate of up to 60 L/min. Because of the widely proved clinical efficacy together with easy application and good patient tolerance in critically ill infants and children, physicians began to focus on the potential roles of HFNC in improving clinical outcomes in adult patients [15]. However, contradictory conclusions were drawn in spite of large number of clinical trials. Fernandez and colleagues conducted a multicenter randomized controlled trial (RCT) in 155 high-risk non-hypercapnic patients and they found that HFNC could not decrease rate of reintubation compared with conventional oxygen therapy (COT) (11% vs. 16%, *P* = 0.5) [16]. On the contrary, a recent randomized trial by Hernández demonstrated that, compared with COT, HFNC could reduce the reintubation rate among extubated patients at low-risk (4.9% vs. 12.2%, *P* = 0.004) [17].

Therefore, based on the disputed findings of HFNC in adult patients after extubation, we assumed that in terms of rate of reintubation, HFNC might be more effective than COT and might be a replacement of NIPPV. We conducted a meta-analysis of all published trials containing superiority test with COT or non-inferiority test with NIPPV, and aimed for identifying the impact of HFNC in improving the outcomes of patients after extubation.

## Methods

### Search strategies

From 1946 to July 2017, a comprehensive computer search was conducted in Pubmed, Embase, Medline, Cochrane Central Register of Controlled Trails (CENTRAL) and Information Sciences Institute (ISI) Web of Science using the keywords of “HFNC” or “high-flow nasal cannula” or “high-flow oxygen therapy” or “nasal high-flow oxygen therapy” and “NIPPV” or “non-invasive positive pressure ventilation” or “noninvasive positive pressure ventilation” or “non-invasive ventilation” or “noninvasive ventilation” or “oxygen therapy” or “COT” or “venturi mask” and “extubation” or “postextubation” without limitation in the publication type or language. We also reviewed the references listed in each identified article and manually searched the related articles to identify all eligible studies and minimize the potential publication bias.

### Inclusion and exclusion criteria

Eligible clinical trials were identified based on the following criteria: 1) the subjects enrolled in each study included patients after extubation; 2) patients were divided into experimental group, in which HFNC oxygen therapy was applied, and control group, in which patients assigned to receive NIPPV or COT; 3) outcomes contained but not limited to mortality, rate of reintubation, length of stay (LOS) in ICU. We excluded studies if they were performed in animals or in patients less than 18 years old, or published as reviews or case reports.

### Study selection

Two independent investigators (He Yu and DL) performed the study selection in two phases. Firstly, they discarded duplicated and non-controlled studies by screening titles and abstracts. Secondly, eligible studies were extracted by reviewing full texts in accordance with the previously designed study inclusion criteria. Any disagreement was solved by mutual consensus in the presence of a third investigator(YN-Ni).

### Data extraction

Independently, two data collectors extracted and recorded desirable information of each enrolled study in a standard form recommended by Cochrane, [18] which consisted of authors, publication year, study design, country, NCT No., population, demographic characteristics (age, gender, etc.), disease conditions (The Acute Physiologic and Chronic Health Evaluation II (APACHE II) and Simplified Acute Physiologic Score II (SAPS II)), outcome measures, and study results. For any missing data information, corresponding authors were contacted by email to request the full original data. Different opinions between the two collectors were determined by reaching a consensus or consulting a third investigator.

### Quality assessment

For the assessment of risk of bias in estimating the study outcomes, we used the Cochrane risk of bias tool [18]. Each study was assessed for: 1) random sequence generation (selection bias); 2) allocation concealment(selection bias); 3) blinding of participants and personnel (performance bias); 4) blinding of related outcomes assessment (detection bias); 5) incomplete outcome data (attrition bias); 6) selective reporting (reporting bias); and 7) other biases. Two investigators conducted the quality assessment for the study methodology, independently and separately. Any divergence was resolved by mutual consensus in the presence of a third investigator.

### Statistical analysis

Statistical analysis of our study was accomplished by an independent statistician using Cochrane systematic review software Review Manager (RevMan; Version 5.3.5; The Nordic Cochrane Centre, The Cochrane Collaboration, Copenhagen, 2014). We used *Mann-Whitney U-test* to verify hypothesis and rendered statistical significance as a Z-value and *P*-value <0.05, and the results were displayed in Forest plots. Continuous variables were reported as mean and standard derivation (SD), while dichotomous variables were shown as frequency and proportion. An initial test for clinical, methodological and statistical heterogeneities was conducted, and we used the χ^2^ test with *P* < 0.1 and I^2^ > 50% to indicate significance. We also performed the sensitivity analysis to substitute alternative decisions or ranges of values for decisions that were arbitrary or unclear. Random-effects model was applied in the presence of statistical heterogeneity; otherwise, fixed effects model was used; for continuous data we calculated mean difference (MD) and 95% confidence interval (CI), while for dichotomous data we calculated odds ratio (OR) and 95% CI.

## Results

**Fig. 1:**
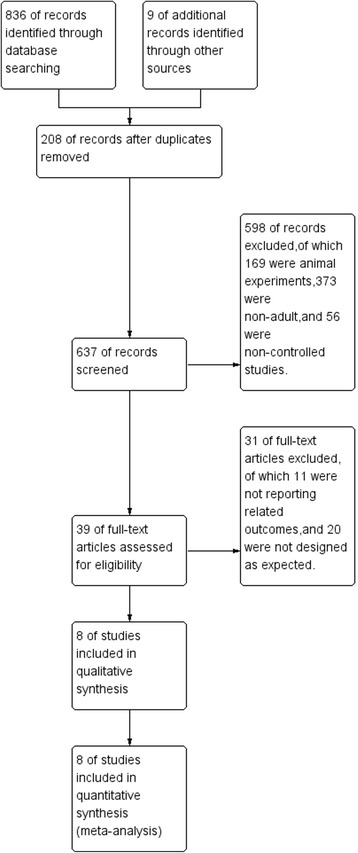
Study flow diagram

Initially 845 records were identified, of which 836 were extracted from electronic databases and 9 were extracted from reference lists review (Fig. 1). By screening the titles and abstracts, 806 studies were discarded for duplication (*n* = 208), animal experiments (*n* = 169), non-adult patients (*n* = 373), and non-controlled studies (*n* = 56). We searched the full-text articles for the remaining 39 studies, and eventually 8 trials [16, 17, 19–24] were enrolled in our final analysis due to 11 studies were not reporting related outcomes, and 20 were not designed as expected.

### Study description

All 8 studies compared the outcomes of HFNC alone with those of NIPPV or COT as a first-line therapy. Mortality was reported in 7 studies [16, 18–21], rate of reintubation was recorded in 8 studies [16, 17, 20–24], and ICU LOS was presented in 7 studies [16, 17, 20–24]. All the studies were RCTs [15, 16, 18–21] Three studies enrolled the patients after cardiac surgery [19, 23, 24], 1 studies enrolled the patients after abdominal surgey [20], 2 studies enrolled the medical patients [20, 22] and 2 studies enrolled both medical and surgical patients [17, 21]. Details of each study were summarized in Table 1.

**Table 1:** Details of the eight enrolled studies

Author(Year)	Study design	NCT No.	Country	Control	Population	Underlying disease	Outcome^a^
Corley 2015	Randomised controlled trial	ACTRN12610000942055	Australia	Standard oxygen therapy	155	Cardiac surgery	②③
Fernández 2017	Randomised controlled trial	NCT01820507	Spain	COT	155	Chronic obstructive pulmonary disease, heart failure	①②③
Futier 2016	Multicenter randomized trial	NCT01887015	France	Standard oxygen therapy	220	Major abdominal surgery	①②③④
Hernández 2016	Multicenter randomized trial	NCT01191489	Spain	COT	527	Medical,trauma,surgical	①②③④⑤
Hernández 2016 (2)	Multicenter randomized trial	NCT01191489	Spain	NIPPV	604	Medical,trauma,surgical	①②③④⑤
Maggiore 2014	Randomized controlled open-label trial	NCT01575353	Italian	Venturi mask	105	Pneumonia, multiple trauma, atelectasis, shock, cardiogenic pulmonary edema, cardiac arrest, other	①②③⑤
Parke 2013	Pragmatic randomized controlled trial	ACTRN12610000973011	New Zealand	Simple facemask or nasal prongs	340	Cardiac surgery	②③⑥
Stéphan 2016	Multicente,r randomised,noinferiorty trail	NCT01458444	France	NIPPV	830	Cardiothoracic surgery	①②③④⑤⑥⑦

^a^Outcome measures include:①mortality;②rate of endotracheal intubation; ③length of ICU stay;④adverse events;⑤respiratory variables;⑥patient comfort;⑦dyspnea scale

*AECOPD* acute exacerbation of chronic obstructive pulmonary disease; *ARDS* acute respiratory distress syndrom; *ARF* acute respiratory failure; *COPD* chronic obstructive pulmonary disease;*COT* conventional oxygen therapy; *HFNC* high flow nasal cannula; *NIPPV* noninvasive positive pressure ventilation; *NR* not report; *SIRS* systemic inflammatory response syndrome

A total of 2936 patients were pooled from all the included trials in our final and meta-analysis, among which 1457 patients were treated with HFNC, 730 patients received NIPPV, and 749 patients used COT. The mean age ranged from 51 to 69 years old. Details of baseline characteristics of patients in each enrolled study were shown in Table 2.

**Table 2:** Baseline characteristics of patients

	HFNC				Control			
Author (Year)	Age,Years Mean (SD)	Man n,(%)	SAPS II Mean(SD)	APACHE II Mean(SD)	Age,Years Mean(SD)	Man, n,(%)	SAPS II, Mean(SD)	APACHE II, Mean(SD)
Corley 2015	63(11.4)	58(72.0%)	NR	15(3.6)	65(11.1)	56(76.0%)	NR	15.0(3.9)
Fernandez 2017	67.3(12.1)	46(59.0%)	NR	11(5.5)	69.7(13.0)	55(71.0%)	NR	10.0(6.7)
Futier 2016	62(12.0)	61(56.0%)	NR	NR	61.0(13.0)	64(57.0%)	NR	NR
Hernández 2016	51(13.1)	164(62.1%)	NR	7	51.8(12.2)	153(58.2%)	NR	7
Hernández 2016(2)	64.6(15.4)	186(64.1)	NR	11	64.4(15.8)	202(64.3)	NR	10
Maggiore 2014	64(17.0)	33(62.3%)	44(16)	NR	65.0(18.0)	35(67.3%)	43.0(14.0)	NR
Parke 2013	65	129(76.3%)	NR	NR	66.0	129(75.4%)	NR	NR
Stéphan 2016	63.8	273(65.9%)	29	NR	63.9	278(66.8%)	28.8	NR

*APACHE* The Acute Physiologic and Chronic Health Evaluation; *COT* conventional oxygen therapy; *HFNC* high flow nasal cannula; *NIPPV* noninvasive positive pressure ventilation; *NR* not report; *SAPS* Simplified Acute Physiologic Score; *SD* standard derivation; *SIRS* systemic inflammatory response syndrome

### Quality assessment

**Fig. 2:**
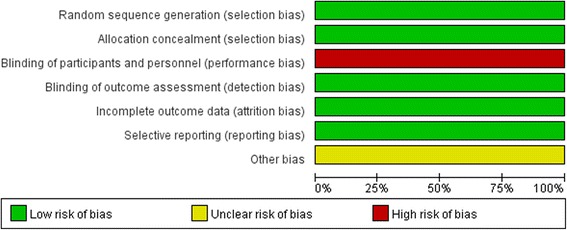
Risk of bias graph

**Fig. 3:**
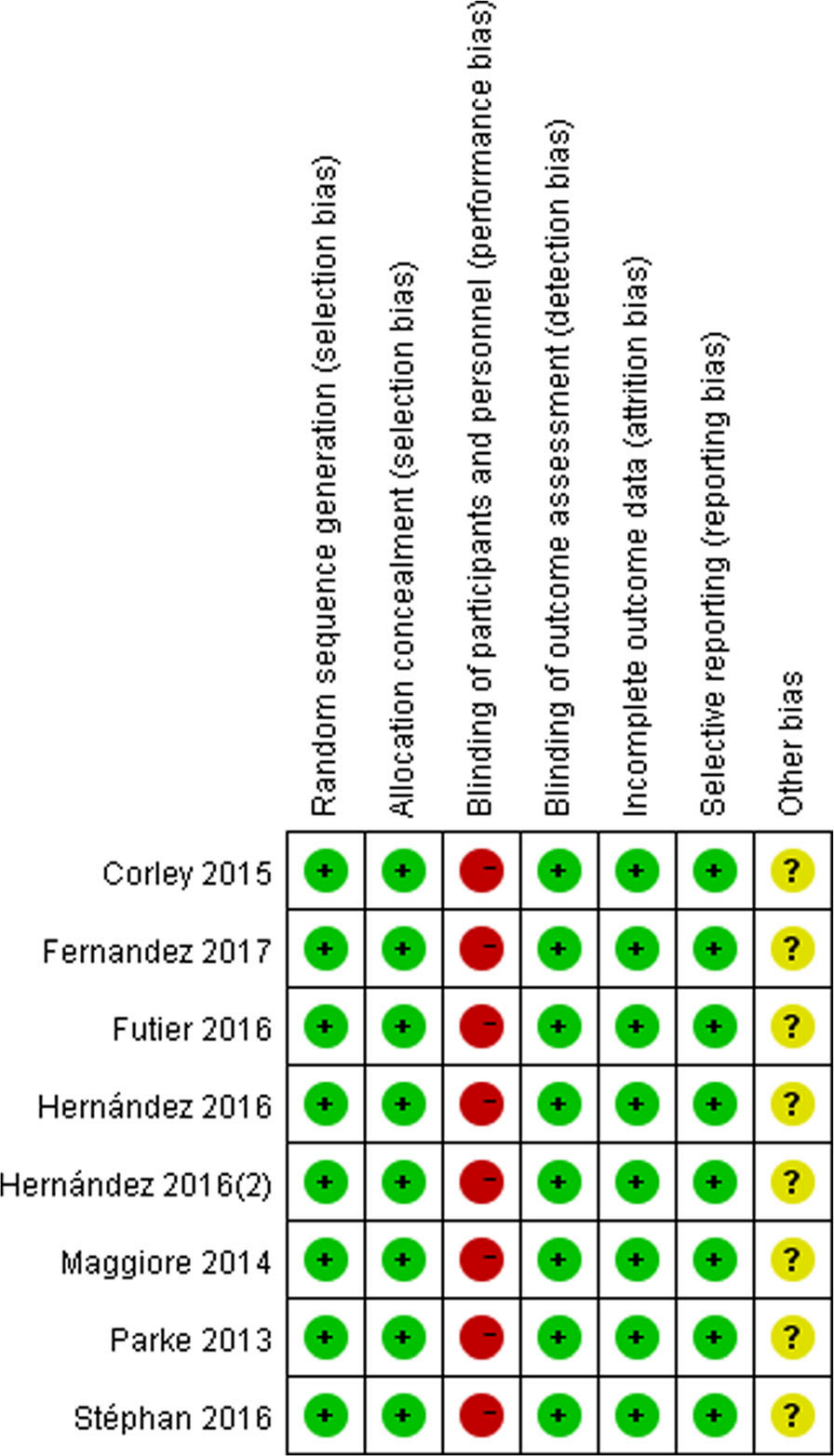
Risk of bias summary

**Fig. 4:**
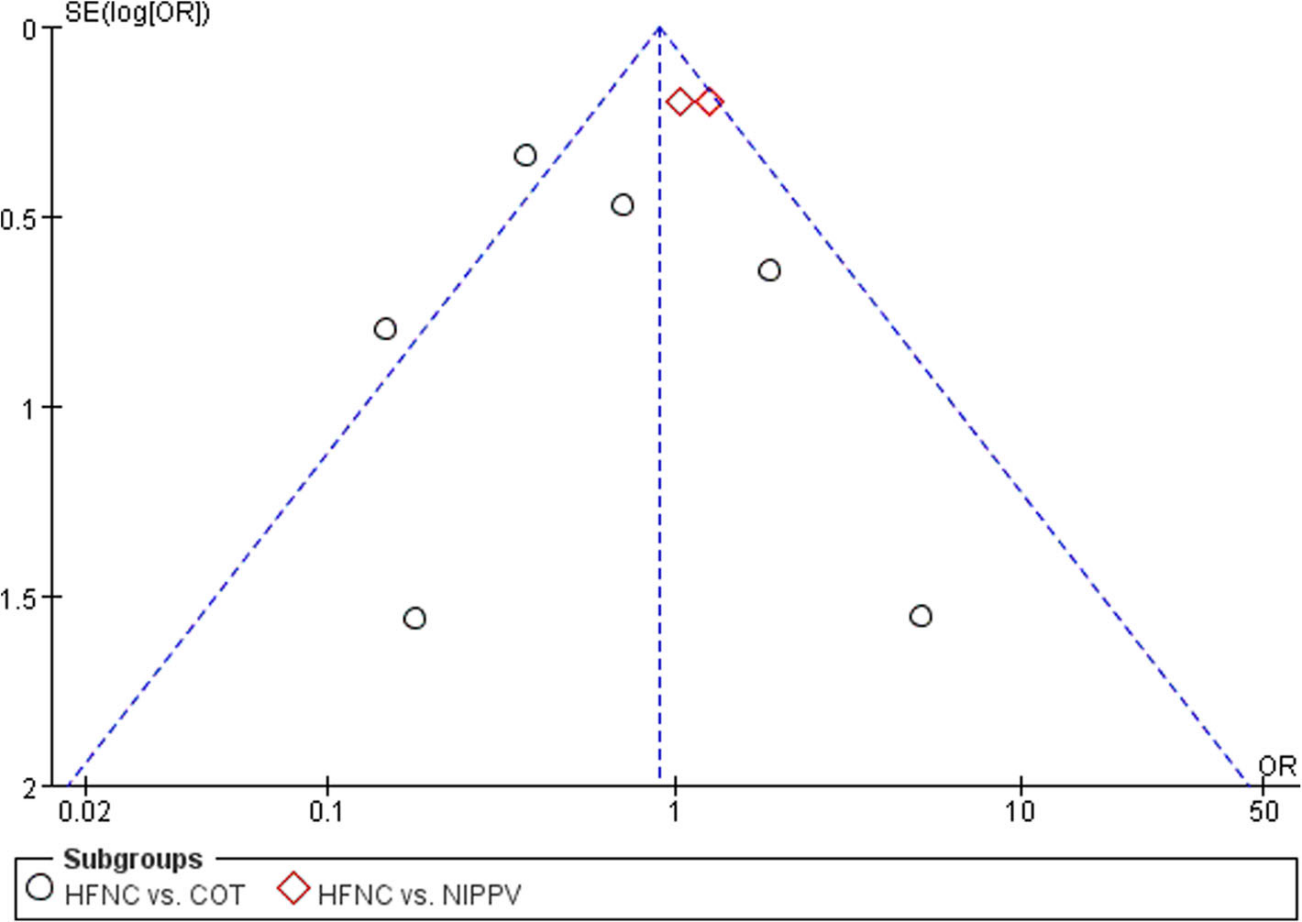
Fig. 4 Funnel plot for publicationbias

Quality assessment of the 6 enrolled studies showed that there was no bias in attribution, detection or reporting in 6 studies, but high bias existed in performance because blinding of patients and personnel seemed to be impossible in any study due to virtual practice issues. (Figs. 2 and 3) No studies were excluded for low quality or dubious decisions in the sensitivity analysis. The publication bias was not found (Fig.4).

### Heterogeneity

Except for the rate of reintubation between HFNC and COT group (I^2^ = 52%, χ^2^ = 10.51, *P* = 0.06), no statistical heterogeneity was found between HFNC and NIPPV group (I^2^ = 0%, χ^2^ = 0.47, *P* = 0.49), in the ICU mortality between HFNC and COT (I^2^ = 0%, χ^2^ = 0.31, *P* = 0.96), or between HFNC and NIPPV (I^2^ = 0%, χ^2^ = 0.03, *P* = 0.87), and in the ICU LOS between HFNC and COT or NIPPV (HFNC vs. COT: I^2^ = 23%, χ^2^ = 5.21, *P* = 0.27; HFNC vs. NIPPV: I^2^ = 0%, χ^2^ = 0.11, *P* = 0.75).

### Rate of reintubation

**Fig. 5:**
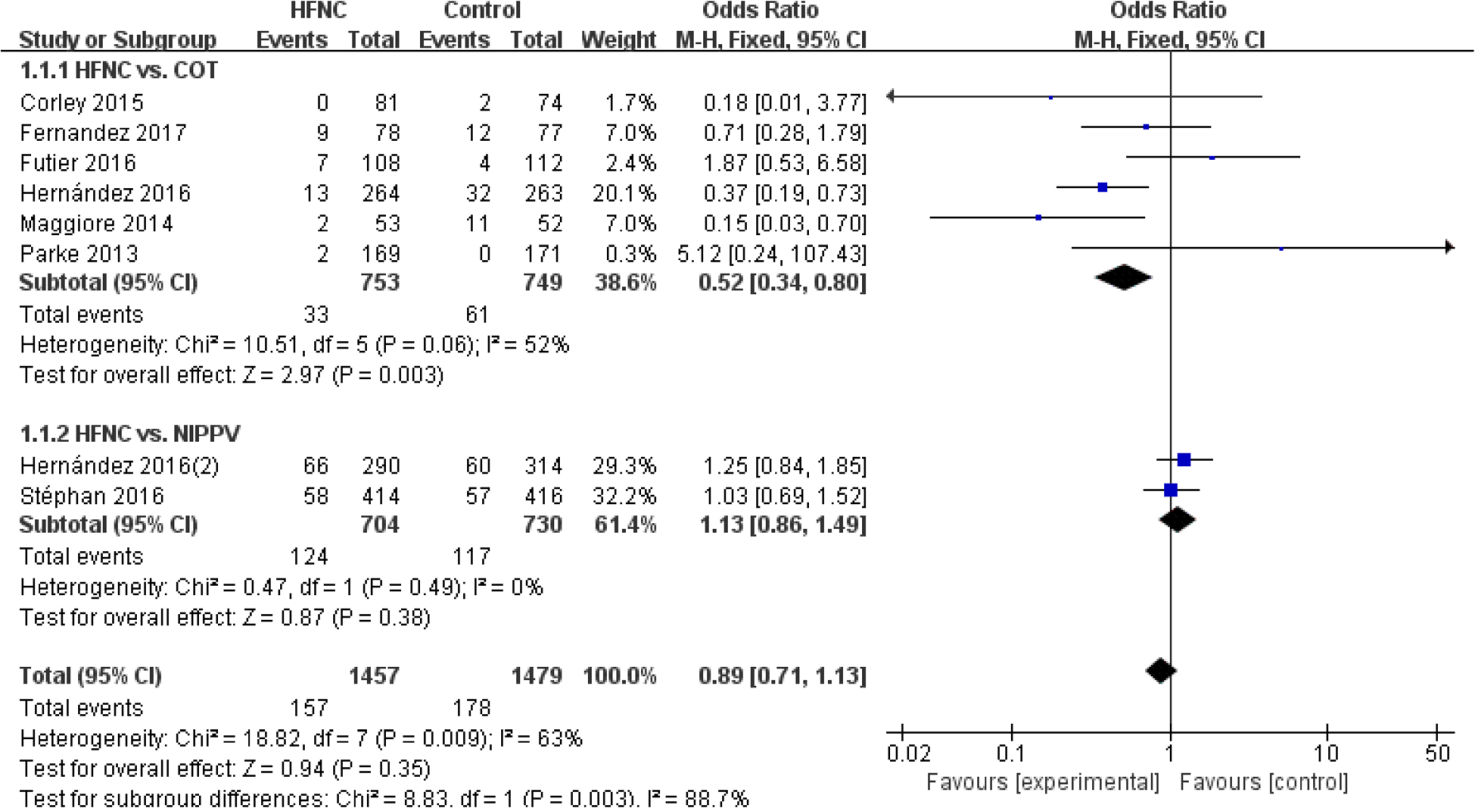
Rate of reintubation. COT, conventional oxygen therapy; CI, confidence interval; HFNC, high-flow nasal cannula; ICU, intensive care unit; NIPPV, noninvasive positive pressure ventilation; SD, standard derivation

Significant difference in the endotracheal intubation was found in HFNC treatment compared with COT (OR 0.52, 95% CI 0.34~0.80, Z = 2.97, *P* = 0.003), but not in the comparison with NIPPV (OR 1.13, 95% CI 0.86~1.49, Z = 0.87, *P* = 0.38) as well as in overall effects (OR 0.89, 95% CI 0.71~1.13, Z = 0.94, *P* = 0.35) (Fig.5).

### ICU mortality

**Fig. 6:**
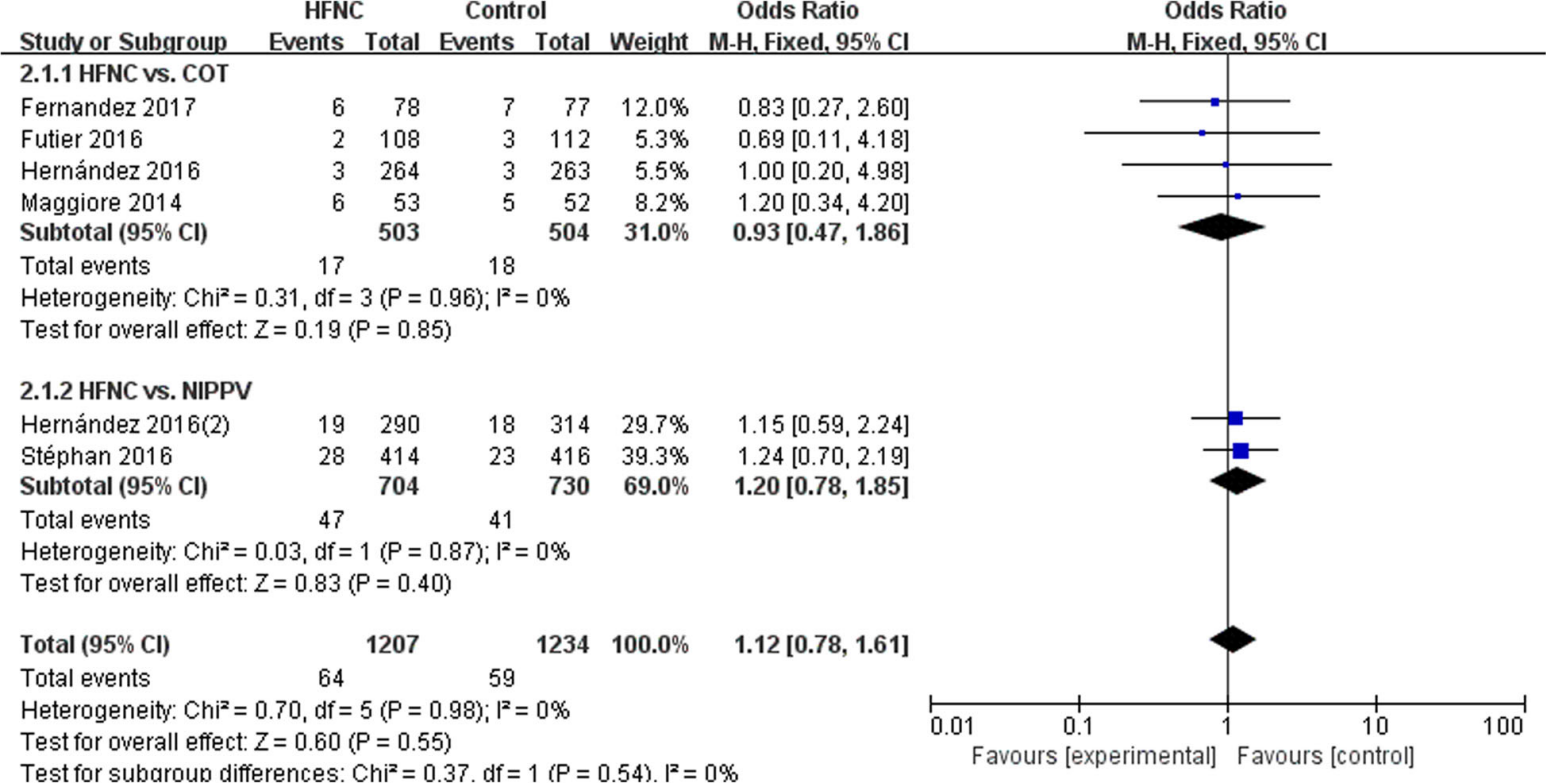
ICU mortality. COT, conventional oxygen therapy; CI, confidence interval; HFNC, high-flow nasal cannula; NIPPV, noninvasive positive pressure ventilation; SD, standard derivation

We did not find significant difference in ICU mortality between treatment with HFNC and COT (OR 0.93, 95% CI 0.47~1.86, Z = 0.19, *P* = 0.85) or NIPPV (OR 1.20, 95% CI 0.78~1.85, Z = 0.83, *P* = 0.40), nor in overall effects (OR 1.12, 95%CI 0.78~1.61, Z = 0.60, *P* = 0.55) (Fig.6).

### ICU los

**Fig. 7:**
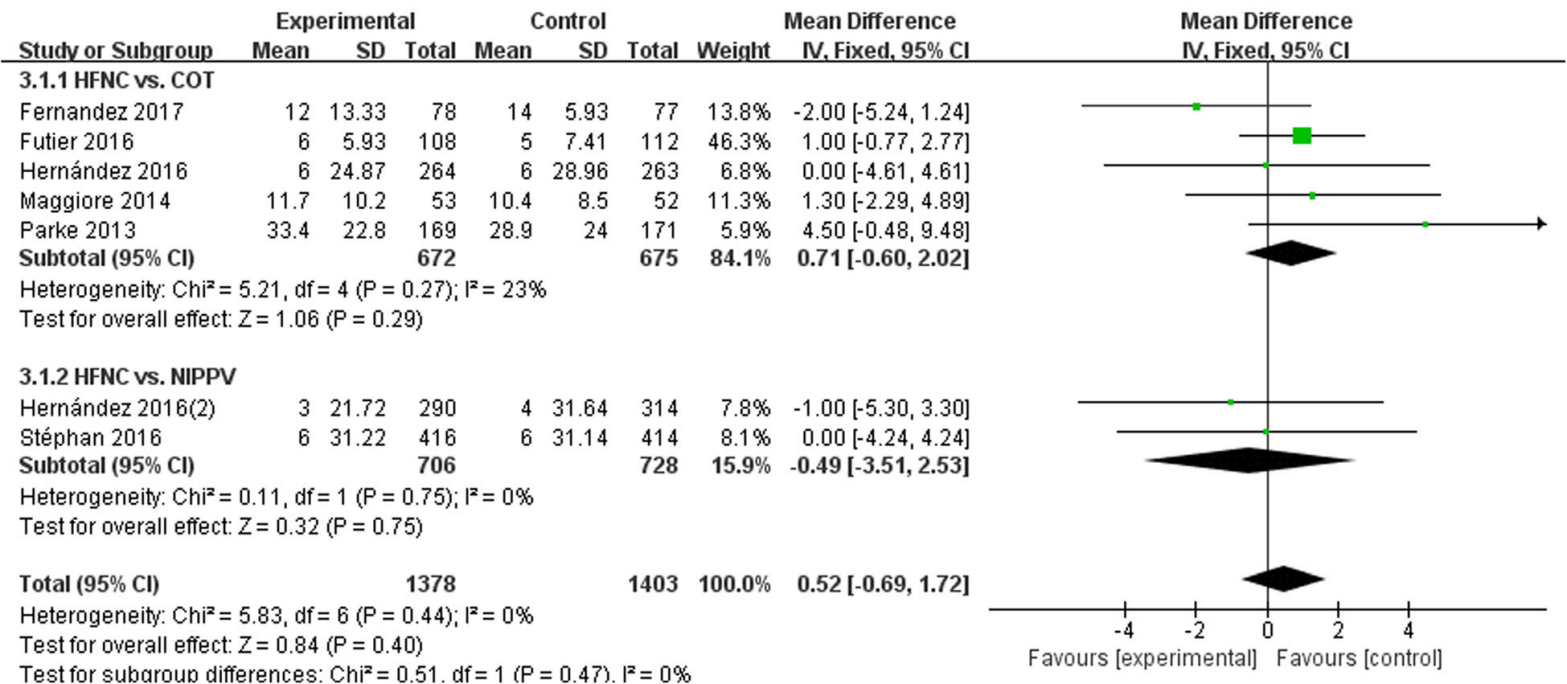
ICU LOS. COT, conventional oxygen therapy; CI, confidence interval; HFNC, high-flow nasal cannula; ICU, intensive care unit; LOS, length of stay; NIPPV, noninvasive positive pressure ventilation; SD, standard derivation

Figure 7 showed that differences of ICU LOS were not significant between HFNC and COT (OR 0.71, 95% CI -0.60~2.02, Z = 1.06, *P* = 0.29) or NIPPV (OR -0.49, 95%CI -3.51~2.53, Z = 0.32, *P* = 0.75), nor in overall effects (OR 0.52, 95%CI -0.69~1.72, Z = 0.84, P = 0.4).

## Discussion

We conducted the meta-analysis to compare the impact of HFNC, COT and NIPPV on rate of reintubation, ICU mortality and ICU LOS. The results showed that HFNC could decrease the rate of reintubation in adult patients after extubation compared with COT, which was equivalent to NIPPV. However, it could not decrease the ICU mortality or the ICU LOS .

HFNC is an excellent oxygen therapy due to its appealing capacity in easy usage, good tolerance and oxygenation improvement [15, 17]. However, no definite conclusions could be drawn because of the inconsistent findings in different studies, which results in our pooled analysis to determine roles of HFNC in improving the outcomes of patients after extubation. In our study, compared with those who used COT, a significant lower need of reintubation in patients after extubation receiving HFNC was presented. The mechanisms of the lower rate of reintubation can be explained as the followings. First of all, as mechanical ventilation will leads to atelectasis even after extubation [25, 26], a positive and expiratory pressure(PEEP) (2-5cmH_2_O) generated by high flow can lead to continuous alveolar recruitment, reduction of airway collapse and improvement of the ventilation-perfusion mismatch [27–30]. Hence larger end expiratory lung volume was observed in patients with HFNC than COT [31]. In addition, the heated humidification closely to physiological conditions preserves the mucosal function and facilitates secretion clearance, thereby further decreasing the risk of atelectasis and improving the oxygenation [32]. Second, adequate minute ventilation and sufficient oxygenation guaranteed by HFNC via delivering a continuous high flow of oxygen accompanied with a higher tidal volume and improved inspiratory flow dynamics [33–35]. Thereby HFNC can decrease respiratory rate and work of breathing [36]. Third, potential pharyngeal dead space washout effect results in a faster decrease of the carbon dioxide and thus a greater fraction of minute ventilation participates in gas exchange [37, 38]. Last, contrary to the unstability FiO_2_ delivered by COT because the entertainment of room air and dilution of the inspired oxygen, constant concentration of oxygen can be delivered to patients due to the high flow and oxygen reservoir within the upper airway [39]. Thus, the risk of insufficient oxygen flow supply for patients, which is common in COT, could be reduced in HFNC [40, 41].

The results of our study were totally different from the previous analyses [42–44], which found no different between HFNC and COT. First of all, we included most recent studies. Second, we only focus on the patients after extubation and surgery, while the other two analyses also included the patients before MV [42, 44]. The respiratory failure often resulted from the initial disease which often had been solved in the patients after extubation. On the contrary, patients after surgery or extubation need respiratory support mainly because of low cough strength and level of consciousness [5, 45]. Third, we only included RCTs, and in the previous studies, non-RCTs were also included.

However, our meta-analysis also indicated that HFNC could not further decrease ICU mortality or ICU LOS compared with COT. As we know, except for respiratory status, numerous factors, especially the concomitant complications such as the acute liver injury and cardiac impairment, may contribute to mortality and ICU LOS [46, 47]. Moreover, it is undeniable that medical resources as well as expenditures are tightly related to the disease outcomes, such as bed availability in general wards and insurance status, which in some extent may offset the positive effects of HFNC.

It has been reported that NIPPV could improve oxygenation and ventilation as well as reduce the risk of respiratory failure in patients after extubation [8–13]. It is believed that NIPPV possessed potential benefits to provide a relatively consistent and wider range of FiO_2_ compared with COT [48]. Moreover, NIPPV could also create an extrinsic PEEP to recruit the collapsed alveoli [49–51]. However, as mentioned above, the limitations of NIPPV due to the adverse events continuously urge physicians and researchers to explore and refine a new oxygen delivery system to prevent potential compromises induced by NIPPV but preserve similar efficiency.

Based on precedent evidence that NIPPV could reduce the rate of intubation in patients after extubation compared with COT [8, 52] as well as similar findings in HFNC in our study, we performed a non-inferiority test between HFNC and NIPPV to further elucidate the potential clinical implications of HFNC. In our meta-analysis, compared with NIPPV, HFNC did not increase the rate of reintubation, which we considered to be attributed to similar effects on respiratory mechanics and gas exchange like providing accuracy FiO_2,_ extrinsic PEEP and guaranteeing sufficient minute ventilation [28–30, 32, 40, 49–51]. Moreover, a lot of studies also reported some advantages of HFNC compared with NIPPV. For example, HFNC can be better tolerated than NIPPV because of more comfortable resulted from a stable flow with warm and humidified gas to reduce the sense of dryness and facilitate secretion clearance [53, 54]. At the same time, the patient-ventilator interaction interface of HFNC was more friendly and would not disturb speaking or eating [55]. Therefore, we might conclude that HFNC is a good replacement of NIPPV in patients after extubation.

However, some clinical heterogeneity existed in our analysis: 1) different end point. Both of the following up time and the primary endpoint were varied among the studies. All of these differences caused the heterogeneity; 2) Heterogeneous treatment strategies. The flow of HFNC and the length of the usage were not unified among studies; 3) Mixed patients. The reasons of MV before extubation were different in enrolled studies including the medical problems and surgery. Moreover, the risks of reintubation were varied in different studies.

Our study has some limitations. Firstly, bias cannot be completely ruled out because blinding was not feasible. Second, the flow of HFNC, the length of HFNC using time after extubation, oxygen therapy interface, end point and following up time were different in our enrolled studies, which may further impede the clinical practice. Third, the underlying diseases of patients in our study were mixed including the ones after surgery and extubation, and even the risk of reintubation was varied. Thus, the second and third limitations contribute to the statistic and clinical heterogeneity of our analysis, which would influence the application of our study. Therefore, future studies are still necessary to further establish standardized application protocols.

## Conclusions

Compared with COT, HFNC could reduce rate of reintubation in patients after extubation, in spite of no benefit in ICU mortality or ICU LOS. It can be considered as a reliable substitute of NIPPV due to similar respiratory mechanics and equivalent clinical outcomes but better compliance and fewer complications.

## Abbreviations

APACHE: The Acute Physiologic and Chronic Health Evaluation
ARDS: Acute respiratory distress syndrome
ARF: Acute respiratory failure
CENTRAL: Cochrane Central Register of Controlled Trails
CI: Confidence interval
COT: Conventional oxygen therapy
FiO_2_: Fraction of inspired oxygen
HFNC: High flow nasal cannula
ICU: Intensive care unit
ISI: Information Sciences Institute
LOS: Length of stay
MD: Mean difference
NIPPV: Noninvasive positive pressure ventilation
OI: Oxygen index
OR: Odds ratio
PaO_2_: Partial pressure of arterial oxygen
PEEP: Positive end expiratory pressure
RCT: Randomized controlled trial
SaO_2_: Arterial oxygen saturation
SAPS: Simplified Acute Physiologic Score
SD: Standard derivation
